# Using expert-elicitation to deliver biodiversity monitoring priorities on a Mediterranean island

**DOI:** 10.1371/journal.pone.0256777

**Published:** 2022-03-24

**Authors:** J. Peyton, M. Hadjistylli, I. Tziortzis, E. Erotokritou, M. Demetriou, Y. Samuel, V. Anastasi, G. Fyttis, L. Hadjioannou, C. Ieronymidou, N. Kassinis, P. Kleitou, D. Kletou, A. Mandoulaki, N. Michailidis, A. Papatheodoulou, G. Payiattas, D. Sparrow, R. Sparrow, K. Turvey, E. Tzirkalli, A. I. Varnava, O. L. Pescott

**Affiliations:** 1 UK Centre for Ecology & Hydrology, Wallingford, United Kingdom; 2 Department of Agriculture, Ministry of Agriculture, Rural Development and Environment, Lefkosia, Cyprus; 3 Water Development Department, Ministry of Agriculture, Rural Development and Environment, Lefkosia, Cyprus; 4 Department of Environment, Ministry of Agriculture, Rural Development and Environment, Lefkosia, Cyprus; 5 Department of Biological Sciences, University of Cyprus, Lefkosia, Cyprus; 6 Oceanography Centre, University of Cyprus, Lefkosia, Cyprus; 7 Terra Cypria - The Cyprus Conservation Foundation, Lefkosia, Cyprus; 8 BirdLife Cyprus, Nicosia, Cyprus; 9 I.A.CO Environmental & Water Consultants Ltd., Lefkosia, Cyprus; 10 Enalia Physis Environmental Research Centre, Lefkosia, Cyprus; 11 CMMI – Cyprus Marine and Maritime Institute, Larnaca, Cyprus; 12 Game and Fauna Service, Ministry of Interior, Lefkosia, Cyprus; 13 Marine & Environmental Research (MER) Lab, Lemesos, Cyprus; 14 School of Biological and Marine Sciences, University of Plymouth, Plymouth, United Kingdom; 15 Department of Maritime Transport and Commerce, Frederick University, Lemesos, Cyprus; 16 Department of Agricultural Sciences, Biotechnology and Food Science, Cyprus University of Technology, Lemesos, Cyprus; 17 Department of Fisheries and Marine Research, Ministry of Agriculture, Rural Development and Environment, Lefkosia, Cyprus; 18 Cyprus Dragonfly Study Group, Pafos, Cyprus; 19 School of Pure and Applied Sciences, Open University of Cyprus, Nicosia, Cyprus; 20 Department of Biological Applications and Technology, University of Ioannina, Ioannina, Greece; University of Pavia, ITALY

## Abstract

Biodiversity monitoring plays an essential role in tracking changes in ecosystems, species distributions and abundances across the globe. Data collected through both structured and unstructured biodiversity recording can inform conservation measures designed to reduce, prevent, and reverse declines in valued biodiversity of many types. However, given that resources for biodiversity monitoring are limited, it is important that funding bodies prioritise investments relative to the requirements in any given region. We addressed this prioritisation requirement for a biodiverse Mediterranean island (Cyprus) using a three-stage process of expert-elicitation. This resulted in a structured list of twenty biodiversity monitoring needs; specifically, a hierarchy of three groups of these needs was created using a consensus approach. The most highly prioritised biodiversity monitoring needs were those related to the development of robust survey methodologies, and those ensuring that sufficiently skilled citizens are available to contribute. We discuss ways that the results of our expert-elicitation process could be used to support current and future biodiversity monitoring in Cyprus.

## Introduction

The earth’s climate and habitats are changing at unprecedented rates, with species and ecosystems increasingly threatened by multiple, often interacting, anthropogenic pressures [1]. Five main threats to biodiversity were identified by the Intergovernmental Science-Policy Platform on Biodiversity and Ecosystem Services (IPBES): changes in land and sea use; direct exploitation of organisms; climate change; pollution; and, invasive alien species (IAS) [1]. The Mediterranean basin is one of the 34 global biodiversity hotspots, including a large number of endemic species, where, due to increased anthropogenic pressure, there is an urgent need for species and habitat conservation [2]. Mediterranean-climate areas are predicted to be likely to experience the highest levels of biodiversity change by 2100 [3]. The most conspicuous threats that affect the greatest number of taxonomic groups in the Mediterranean are habitat loss and degradation, followed by the unsustainable exploitation of species, pollution, climate change, eutrophication and species invasions [4]. Increased tourism and urbanisation in this region also adversely affect biodiversity through habitat loss, disturbance, use of pesticides and herbicides and increased pollution [5–7]. In addition, over-exploitation of natural resources is specifically an issue for fisheries of the Mediterranean Sea [8].

To attempt to halt and, where possible, reverse species population declines and habitat degradation, policy-makers need to use scientifically robust biodiversity data [9, 10]. Monitoring is therefore needed to provide evidence on biodiversity status and trends [11]. Hochkirch and others [12] suggest an eight-point strategy for reducing gaps in available biodiversity data. At the local level, the most relevant of these are: increasing explorative field surveys; increasing monitoring of less well-studied taxa; building capacity in areas where there is high species-richness or levels of endemism; providing mechanisms of funding in order to fill knowledge gaps; and the use of global online data repositories such as the Global Biodiversity Information Facility (GBIF; https://www.gbif.org/) for hosting high quality, open data. Open data can be an important mechanism to enable the effective use of biological monitoring information [13]. The increasing use of technologies for biological recording has resulted in many millions of records being made freely available through global platforms such as GBIF. However, open data repositories may also contain important biases in the records uploaded [12, 14]. The other elements of Hochkirch and others [12] strategy: linking information on taxonomy to information on conservation; the mapping of spatial threat data; and automating pre-assessments, could also be relevant locally, although they are further removed from the basic task of documenting what biodiversity occurs where.

Information on species distributions and identification [15] is essential for supporting conservation [16], but funding and support for taxonomic training may also be limited [17], with numbers of taxonomists decreasing globally [18, 19]. Initiatives such as the Distributed European School of Taxonomy, which offers education and training opportunities (https://cetaf.org/dest/about-dest/), are one mechanism for bridging these gaps between data needs and data availability; however, limited support for both taxonomic training and the availability of employment still present challenges for conservation [20, 21]. Indeed, the “provision of funding mechanisms” was another strategy highlighted by Hochkirch and others [12] as important for increasing the availability of biodiversity data. Technology is also increasingly being used to fill knowledge gaps, as it can assist with the surveying of large or difficult to reach areas more rapidly and cheaply than on-the-ground surveys. For example through remote sensing [22], the use of habitat suitability maps [2], and environmental DNA [23].

In some areas, e.g. the UK, reduced governmental funding for the collection and curation of records within statutory organisations has led to a diversification in mechanisms for biological data gathering [24]. In this situation, citizen science, the involvement of volunteers in generating scientific data, is often seen as an attractive option by policy-makers to fill knowledge gaps in biodiversity datasets [25]. Citizen science can provide broad geographic coverage of species’ occurrences [26, 27] and has been used to monitor trends in the distributions [28, 29] and abundances of species [30–32]. Biodiversity-based citizen science typically follows two methods of data collection: structured biodiversity recording, i.e. recording that follows some sort of protocol or design, and unstructured recording, which refers to the *ad hoc* collection of records [28]. The provision of early warnings of the arrival of new IAS is one area in which this has been successful [33–35]. Even in regions without long-standing traditions of amateur contributions to natural history, important discoveries have been made in this way. For example, a new marine alien species to Cyprus was discovered by recreational divers in 2019, and was identified through photographs posted in the online data repository of the iSea project “Is it Alien to you? Share it!!!” [36].

Despite all of this biodiversity monitoring activity, i.e. the existence of open databases, new technology, and the diversification of mechanisms for collecting monitoring data, knowledge gaps still exist [37, 38]. Where existing data and models cannot answer questions needed to inform management and policy, expert-elicitation, i.e. using the knowledge and experience of experts, can be used to address these gaps [39]. Expert-elicitation methods, such as the Delphi technique and similar approaches [40, 41], have been used to address many questions in conservation science [42], including the prioritisation of knowledge needs for enabling implementation of “nature-based solutions” in the Mediterranean [43], and the prioritisation of needs for biodiversity monitoring in the UK [44]. Prioritising biodiversity monitoring requirements or “needs” for data contributors (whether amateur or professional), and end users of data (e.g. governments), can prove a useful mechanism for mediating between potentially competing priorities between such groups [44], and can help to focus spending where resources are limited.

We use expert-elicitation approaches to collaboratively prioritise the biodiversity monitoring needs of a range of stakeholders, with expertise and/or experience in biodiversity monitoring, on a Mediterranean island (Cyprus). This was undertaken in order to increase understanding of the relative importance of different biodiversity monitoring needs, and to provide a hierarchical checklist on which to base future policy development, resource allocation, and general biodiversity conservation decision-making.

### Biodiversity and monitoring on Cyprus

The island of Cyprus is the third largest island in the Mediterranean [45] and very rich in biodiversity in relation to its size [46]. Cyprus is home to a variety of landscapes, species and habitats of European importance, and has high cross-taxon levels of endemism [47–50]. Plants in particular have one of the highest levels of endemism in the European Union [51, 52].

The Government of the Republic of Cyprus and Sovereign Base Area Administration fund the monitoring of protected species and habitats. However, to date, as throughout the world, the biodiversity recording effort on Cyprus has been uneven, with many geographical areas and taxonomic groups poorly represented on biodiversity data platforms, particularly at finer spatial scales. Whilst structured biodiversity monitoring does exist on the island, this is generally aimed at generating temporal trends in population counts, and is largely restricted to birds; although we also note the monitoring of the endemic mouflon going back over two decades. BirdLife Cyprus has been publishing annual reports of unstructured bird records since 1970, as well as monthly checklists based on reports by birdwatchers. Their longest running structured monitoring scheme is the monthly water bird survey (starting 2005). The Cyprus Dragonfly Study Group and the Cyprus Butterfly Group have also recently begun to contribute data to pan-European projects [53, 54]. Plants have been well-recorded at a broad scale [55], but, finer-scale information, such as might be used to generate “atlas”-style distribution maps [27], and information on composition and change at the plant community scale, appear to be largely absent at the time of writing, although monitoring of Red Data book plants is in place [52]. Habitat mapping and conservation assessment takes place largely within the Natura 2000 network for areas in the Republic of Cyprus and the Sovereign Base Areas. The frequency of monitoring is strongly associated with reporting obligations and available funds. In addition, biodiversity data are occasionally collected as part of research projects and environmental impact studies.

In order to understand the diversity of biodiversity monitoring activity currently in place across Cyprus, and in an attempt to see whether consensus could be reached on the most important monitoring needs across the island, stakeholders from across conservation and ecology reviewed and prioritised these, through both an online questionnaire and an in-person workshop, in August 2017. The workshop followed published methods [44, 56], adhering to the ten guiding principles later published by Roy, Peyton and Booy [41], to generate a prioritised list of biodiversity monitoring needs. This is the first time, to our knowledge, that such an approach for prioritising biodiversity monitoring needs has been undertaken within a mediterranean-climate zone.

## Methods

**Fig 1:**
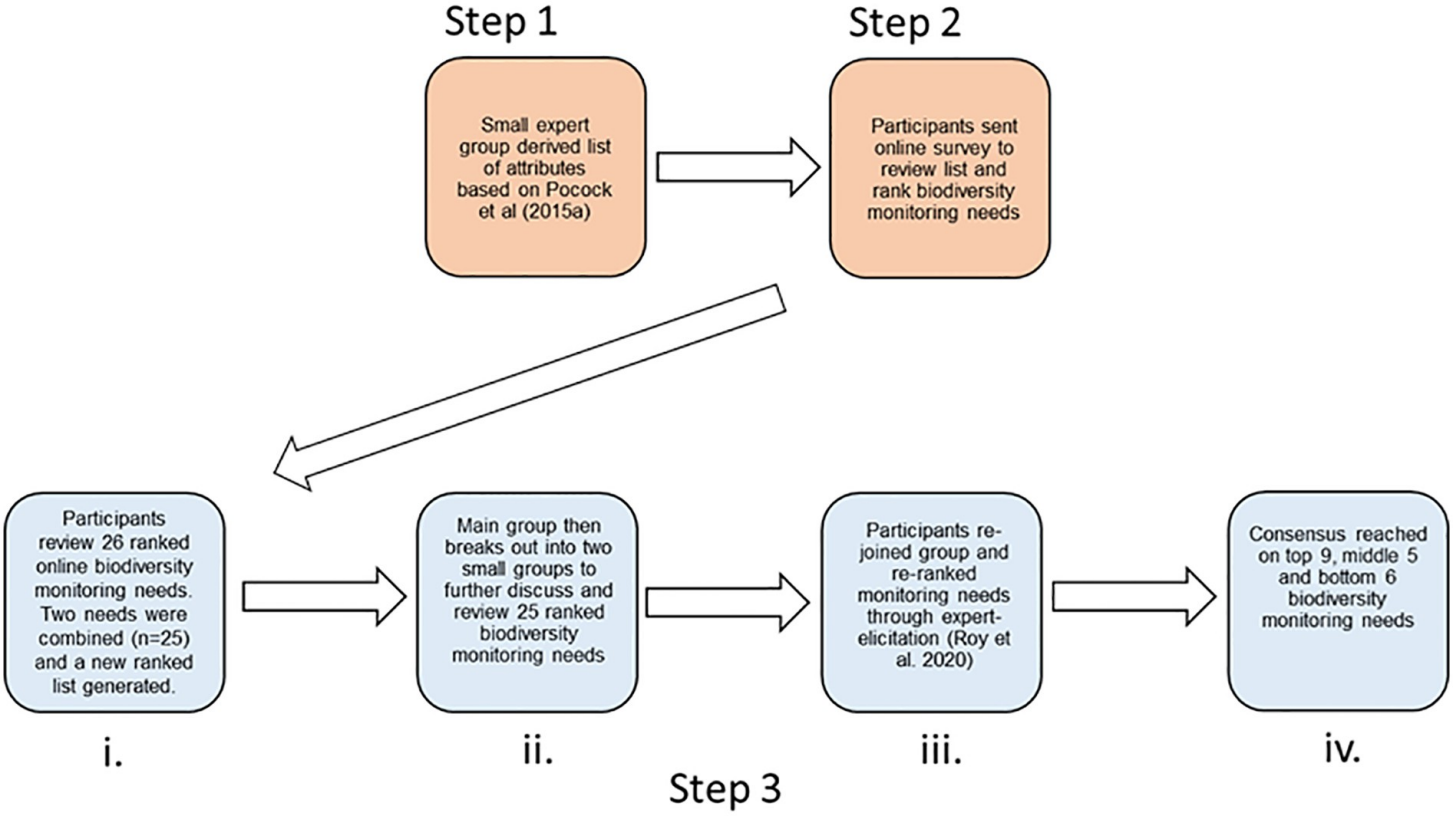
Outline of process to create and prioritise a prioritised consensus list. The process used to create and prioritise a consensus list of biodiversity monitoring needs for Cyprus. Orange boxes denote the work undertaken in advance of the workshop (Step 1–2) and blue boxes denote tasks undertaken during the expert-elicitation workshop in August 2017 (Step 3). Forty-seven stakeholders were invited to take part in Step 2 of the process; 27 took part. Thirty-nine out of 56 invited stakeholders took part in Step 3 of the process. The 39 stakeholders included those asked to take part in Step 2 but a wider pool of stakeholders were also approached to take part as interest in the workshop increased.

The study was designed to develop a list of prioritised biodiversity monitoring needs for Cyprus. Expert-elicitation methods used by Pocock and others [44], to develop a prioritised list of attributes for designing biodiversity monitoring programmes in the UK, were adapted for use in Cyprus (note that where Pocock and others [44] refer to attributes and needs, we use the term “biodiversity monitoring need” throughout). The expert-elicitation process for this workshop was carried out using a three-step process (Fig 1).

### Step 1: Developing the list of biodiversity monitoring needs in Cyprus

A list of 25 biodiversity monitoring needs, taken from Pocock and others [44], was reviewed and revised by JP (author), Angeliki Martinou and Helen Roy (part of the project team for which the workshop was run) for relevance to Cyprus. The modified list was then shared with stakeholders at the Department of Environment, Government of the Republic of Cyprus for review. After consultation with MH (author) the list was supplemented with an additional monitoring need specific to Natura 2000 sites.

### Step 2: Assessing the importance of the biodiversity monitoring needs for Cyprus

**Fig 2:**
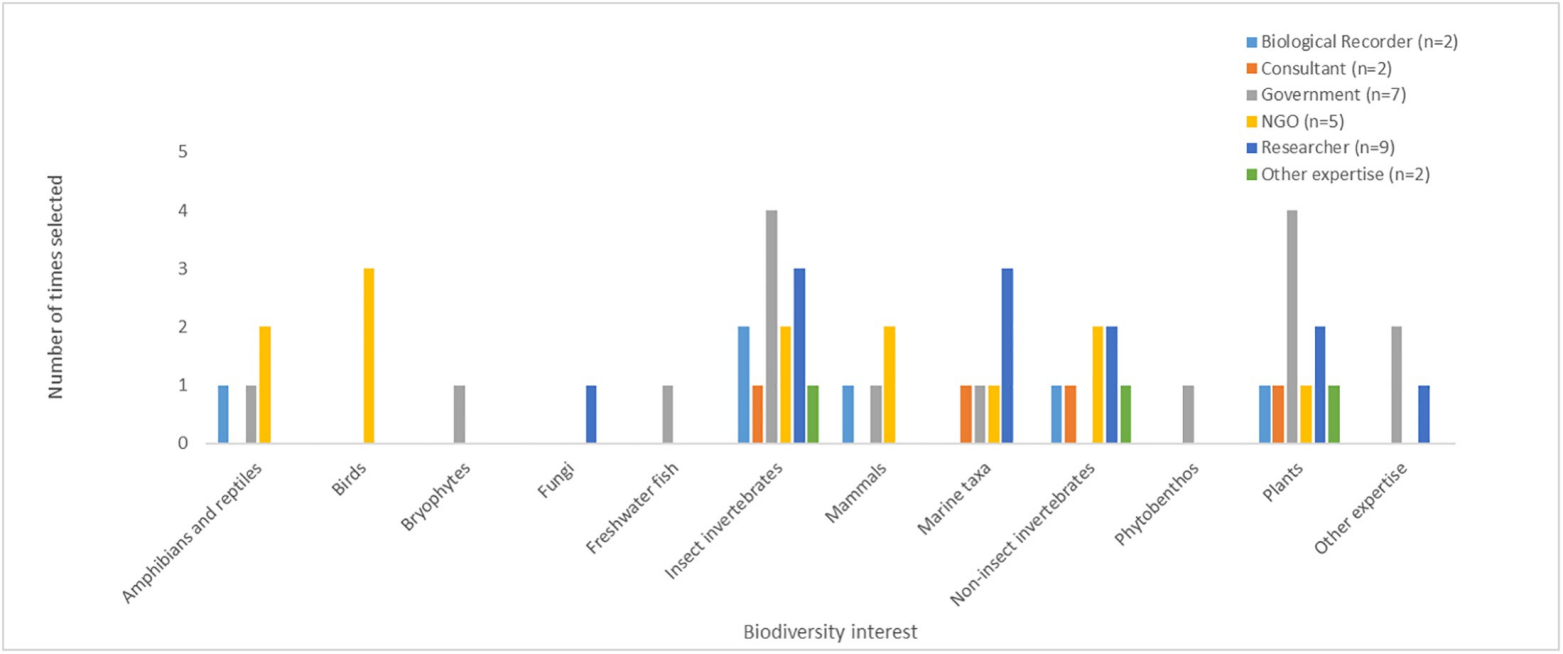
Biodiversity interests of stakeholders. Biodiversity interests of stakeholders working in the field of biodiversity monitoring in Cyprus, who took part in an online questionnaire (Step 2 in the expert-elicitation process); 27 usable responses were received. A pre-populated list of taxonomic interests was given in the question, along with a free text box for additional taxonomic or environment-focused interests. Twenty-three out of the 27 stakeholders selected their top three biodiversity interests; three stakeholders selected more than three biodiversity interests. One stakeholder did not respond and this result was added to “Other expertise”.

A questionnaire (see S1 File), written in both Greek and English, and hosted on the GDPR-compliant survey platform https://www.onlinesurveys.ac.uk, was distributed by email to 47 stakeholders with expertise in recording and/or monitoring biodiversity in Cyprus. These stakeholders either had strategic oversight of monitoring, extensive practical experience, and/or participated in monitoring in a professional or voluntary capacity. Stakeholders included volunteer experts who ran biological recording schemes and coordinated others to gather species records, academics from universities and research institutes, and employees from private research and consultancy organisations. They also included stakeholders involved in data collection from government agencies and non-governmental conservation organizations. Those invited possessed expertise in a wide range of taxa and habitats across Cyprus (Fig 2 and Table 1).

**Table 1:** Results of ranking exercise of biodiversity monitoring needs.

* Theme	Step 2 list of biodiversity monitoring needs (adapted from Pocock et al. [44])	Total number of times selected in top 10	Biological recorder (n = 2)	Consultant (n = 2)	Government (n = 7)	NGO (n = 5)	Researcher (n = 9)	Other expertise (n = 2)
Design	There is standardised methodology and protocols to ensure consistency	18	-	2 (100%)	4 (57%)	4 (80%)	7 (78%)	1 (50%)
Human resource	Mentoring, training and support for contributors is provided	18	2 (100%)	1 (50%)	5 (71%)	3 (60%)	5 (56%)	2 (100%)
Design	There is national or regional co-ordination	17	2 (100%)	1 (50%)	5 (71%)	3 (60%)	4 (44%)	2 (100%)
Human resource	There are quality assurance checks undertaken in order to ensure the accuracy of the records	17	2 (100%)	1 (50%)	4 (57%)	3 (60%)	6 (67%)	1 (50%)
Human resource	There are sufficient contributors with specialist knowledge of their taxa	15	1 (50%)	1 (50%)	4 (57%)	3 (60%)	5 (56%)	1 (50%)
Analytical	There are appropriate analytical/statistical approaches to measure trends from monitoring data	14	-	1 (50%)	2 (29%)	4 (80%)	5 (56%)	2 (100%)
Technological	There are data systems (e.g. online) for efficient data capture and storage	14	1 (50%)	2 (100%)	3 (43%)	2 (40%)	5 (56%)	1 (50%)
Human resource	There is sustained participation	12	2 (100%)	-	2 (29%)	2 (40%)	5 (56%)	1 (50%)
Design/Human resource	There is wide coverage across the country/region, e.g. covering remote and well-populated areas	11	1 (50%)	-	1 (14%)	3 (60%)	5 (56%)	1 (50%)
Design	There are suitable field sampling methods that are accurate/efficient	11	-	1 (50%)	4 (57%)	3 (60%)	2 (22%)	1 (50%)
Other resource	There are suitable and accessible identification guides	11	2 (100%)	1 (50%)	3 (43%)	1 (20%)	3 (33%)	1 (50%)
Analytical	Change is reported at appropriate intervals	10	-	1 (50%)	1 (14%)	2 (40%)	6 (67%)	-
Human resource	There are sufficient contributors	10	2 (100%)	-	5 (71%)	1 (20%)	1 (11%)	1 (50%)
Design	‘Important’ or ‘indicator’ species have been identified	10	-	-	-	3 (60%)	5 (56%)	2 (100%)
Analytical	There is access to analytical expertise to measure trends from monitoring data	10	-	-	3 (43%)	1 (20%)	5 (56%)	1 (50%)
Human resource	There is appropriate feedback to participants on survey results and findings	9	2 (100%)	1 (50%)	2 (29%)	1 (20%)	2 (22%)	1 (50%)
Design	Communicate the objectives of monitoring	9	2 (100%)	2 (100%)	2 (29%)	2 (40%)	1 (11%)	-
Design/Human resource	There is extra effort on protected areas, e.g. Natura 2000 sites	9	-	-	2 (29%)	3 (60%)	3 (33%)	1 (50%)
Design	There is a scientific scheme design (such as stratified or randomised site selection) for statistical rigour	8	-	-	1 (14%)	2 (40%)	3 (33%)	2 (100%)
Analytical	Change is reported on an annual basis	7	-	2 (100%)	1 (14%)	-	3 (33%)	1 (50%)
Design/Human resource	There is extra effort on priority species and habitats	6	1 (50%)	1 (50%)	1 (14%)	2 (40%)	1 (11%)	-
Design/Human resource	Recorders collect supplementary data (such as characteristics of the habitat, soil or weather)	5	-	-	-	1 (20%)	3 (33%)	1 (50%)
Technological/Human resource	The data from monitoring schemes are widely disseminated	5	-	1 (50%)	1 (14%)	-	3 (33%)	-
Design/Technological	There are simple ways for everyone to report widespread/common/easily-identified species	4	-	-	2 (29%)	1 (20%)	1 (11%)	-
Human resource	Examples of best practice are identified and shared between schemes and organisations	4	1 (50%)	-	1 (14%)	1 (20%)	-	1 (50%)
Technological	There are systems for electronically capturing data in the field	2	-	1 (50%)	1 (14%)	-	-	-

Results of ranking exercise of biodiversity monitoring needs from an online questionnaire sent to 47 invited stakeholders working in the field of biodiversity monitoring in Cyprus. Twenty-seven stakeholders ranked their biodiversity monitoring needs for Cyprus from a list of 26 biodiversity monitoring needs. Stakeholders were asked to rank the monitoring needs that represented the 10 most important gaps or opportunities in biological recording in Cyprus, based on their perspective or experience (whereby 1 was the most important gap or opportunity and 10 was the least important gap or opportunity). The number of times each biodiversity monitoring need was selected by all the stakeholders is given alongside it. This score was then broken down into the number of responses from each of the six stakeholder affiliations and the percent of times it was chosen by the stakeholders within that affiliation, e.g. two consultants and they both selected “*There is standardised methodology and protocols to ensure consistency*” = 2 (100%). The cells are coloured in a grey-scale continuum, for the percent chosen from within each of the affiliations: 0%, 1–24%, 25–49%, 50–74% and 75–100%.

*Themes were added post-workshop.

The questionnaire was sent to stakeholders two weeks prior to the workshop and was completed by individuals in private. Stakeholders were asked to select their organisation from a pre-populated list consisting of the following options: NGO; Government employees; University; Research Institute; Biological Recording (i.e., amateur experts, or citizen scientists); and, Other. They were also asked to provide additional information on their background if they selected the “Other” category. Stakeholders then selected their top three biodiversity interests from the list provided. A free-text box allowed the stakeholders to add additional information as needed.

Next, stakeholders were asked to select their top ten monitoring needs from the list of 26 provided (Step 1) and to rank them. Stakeholders were encouraged to undertake this ranking task in a two-step process: (1) first review the list of 26 needs and mark ’N/A’ for the 16 biodiversity monitoring needs that they considered least important, until they were left with the ten that were most important to them; (2) second, assign a score of 1–10 (with 1 being most important and 10 being least) to each of their remaining ten biodiversity monitoring needs. Stakeholders were also invited to add any further comments in a free text box. These comments were to be picked up in the wider discussion session within the expert-elicitation workshop (Step 3). The results of the ranking exercise from each of the 27 stakeholders who responded (out of the 47 contacted) were combined to generate a single ranked list of 26 biodiversity monitoring needs (Table 1).

The final question asked stakeholders to give one overall score as to whether the list of 26 biodiversity monitoring needs adequately represented perceived gaps in Cyprus. Stakeholders were asked to give a score between 1 (not useful at all) to 10 (very useful). S2 File gives the response data received from the 27 stakeholders.

### Step 3: Collaborative prioritisation of an overall list of biodiversity monitoring needs in Cyprus

An expert-elicitation workshop was held on 31st August 2017 for our stakeholder network in order to try and reach a consensus on the top biodiversity monitoring needs (Fig 1). Thirty-nine out of 56 invited stakeholders attended the workshop. These stakeholders were from the same sectors as in Step 2, and were from both the UK and Cyprus; UK participants were either active in biodiversity recording in Cyprus, or were invited to provide their experiences of prioritising biodiversity monitoring needs with limited resources. The 39 stakeholders included some of those who had taken part in Step 2 (n = 16) and also stakeholders who had not (n = 23). The workshop was combined with a plenary session with presentations from all stakeholder groups, in order for the stakeholders to understand the range of monitoring already undertaken across Cyprus, and to learn about potentially relevant UK approaches. An overview presentation of the summary results from the online survey was also given during the plenary session (S2 File).

#### Step 3i

Following the presentation of the online survey results, stakeholders discussed and reviewed the ranked list of 26 biodiversity monitoring needs from the online survey (Step 2) during the plenary. During the discussions, it was agreed that two of the monitoring needs could be merged:

“*There are sufficient contributors with specialist knowledge of their taxa combined*” was merged with “*There are sufficient contributors*”, to give: “*There are sufficient ’and sustained’ contributors with specialist knowledge of their taxa*”.

This then gave 25 biodiversity monitoring needs taken forward for further discussion and ranking in breakout groups.

#### Step 3ii

The group of 39 stakeholders was divided into two breakout groups to discuss and review the ranked list of 25 biodiversity monitoring needs. The breakout groups then re-merged in a final plenary session to review the biodiversity monitoring needs and generate an agreed overall list of these.

#### Step 3iii

The results of the online ranking exercise of biodiversity monitoring needs (S2 File) were re-summarised (Table 1) and used as a point from which to start the consensus-building process. During the exercise it was made clear to stakeholders that each biodiversity monitoring need could be moved up or down the list, with the ultimate location in the list not being directly dependent on the original online rank score. Stakeholders were also encouraged, following the discussions of the breakout groups, to edit the monitoring needs where it was felt they needed combining, or exhibited redundancy, and also to suggest additional needs that were missing from the original list.

#### Step 3iv

The biodiversity monitoring needs were collectively prioritised into three categories during the expert-elicitation process, the top (most important), middle (intermediate importance) and bottom (least important) (Table 3). Stakeholders preferred to represent the results three categories as opposed to an individually ranked list.

**Fig 3:**
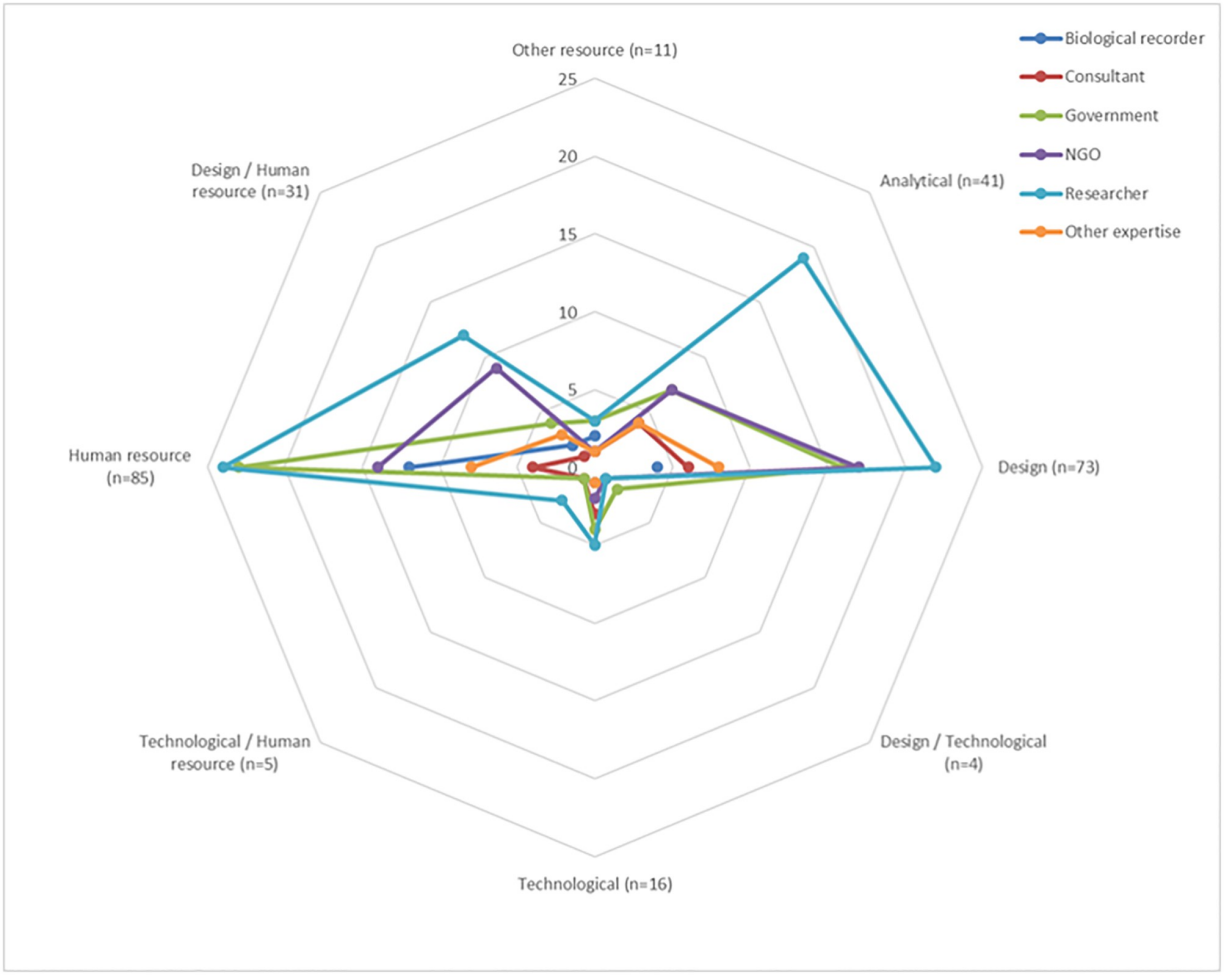
Radar chart showing the results of an online questionnaire. Radar chart showing the results of an online questionnaire sent to 47 stakeholders. The 26 biodiversity monitoring needs were resolved into eight themes post-workshop: Analytical, Design, Design/Technological, Technological, Technological/Human resource, Human resource, Design/Human Resource, and Other resource. The number of times stakeholders selected each of the eight themes is given on the radial axis next to the theme name.

After the workshop, general themes were ascribed to all the biodiversity monitoring needs in order to assess the relative priorities of different types; however, these should not be taken as the only possible categorisation (Tables 1 and 3, Fig 3). Five themes were assigned as follows:

1. Analytical, e.g. the capacity to analyse data to produce biodiversity time trends;
2. Design, e.g. monitoring design for the programme including protocols around reporting of species;
3. Human resource, e.g. the time contribution or availability of the participant or coordinator to ensure that data are collected and collated;
4. Technological, e.g. the use of methods involved in data capture such as data loggers or the use of mobile applications; and
5. Other resource, e.g. any resource that was not considered to fit within the previous themes.

A sixth theme was assigned to the additional comments received as part of the online questionnaire and expert-elicitation workshop, “Political”. This theme was assigned to comments where overarching or management-level decision-making issues were raised. Additional comments raised from the online survey and workshop were brought into the discussions of the expert-elicitation workshop, but these points were not ultimately included in the final list.

The individual themes were combined where biodiversity monitoring needs were considered to have spanned several themes. For example, Design/Human resource for the need: “There is wide coverage across the country/region, e.g. covering remote and well-populated areas” to recognise that this need would require both participant engagement to physically record in different areas, alongside survey design to ensure representative distribution data were collected.

## Results

### Step 1: Developing the list of biodiversity monitoring needs in Cyprus

Following the initial review of the list of 25 biodiversity monitoring needs [44], the text “*There is good retention of contributors*” was modified slightly so as to generate a clearer biodiversity monitoring need: “*There is sustained participation*” (note that biodiversity monitoring needs are given in italics throughout). The modified list was then further amended following input from MH (author) to include the additional biodiversity monitoring need:

“*There is extra effort on protected areas*, *e*.*g*. *Natura 2000 sites*”. This resulted in 26 monitoring needs for inclusion in the questionnaire described below (see Table 1).

### Step 2. Online survey results

#### Stakeholder affiliation

Twenty-nine out of 47 invited stakeholders completed the online questionnaire prior to the workshop; two results were excluded due to not having sufficient data for analysis, leaving 27 records for use in the workshop (S2 File). The 27 stakeholder respondent affiliations were re-categorised post-workshop, based on the information provided, in order to create what were considered to be more informative groups. As such, University and non-private Research Institute were combined to give “Researcher”, and “Consultant” was added to give more detail for affiliations previously listed as “Other”. The following six post-workshop affiliation types were used in the analyses: Biological recorders (n = 2); Consultants (n = 2); Government staff (n = 7); NGO staff (n = 5); Researcher (n = 9); and Other expertise (including non-response) (n = 2).

#### Stakeholder biodiversity interests

Fig 2 provides a summary of the results from stakeholders who responded to the online question asking for information on biodiversity interests. Note that the category “Insect invertebrates” included those with expertise in aquatic invertebrates; also, “inland fish” was renamed “freshwater fish” for clarity. “Other expertise” included non-response data. No environment-focused (as opposed to taxon-focused) responses were given (Fig 2). A maximum of 81 affiliation/biodiversity interest combinations were possible for the 27 respondents (three choices x 27). Fifty-four combinations were returned, with three respondents choosing more than three options. Government and research stakeholders showed a broad range of monitoring interests across taxonomic groups, while the NGO stakeholders focused on amphibians, reptiles and birds. Plants and insect invertebrates (including aquatic taxa) were the best represented taxonomic groups (Fig 2).

#### Top ten priority monitoring needs

The results of the online ranking exercise (Step 2) for the top ten needs are given in Table 1. All 26 monitoring needs, from the original list, were selected at least once by a stakeholder. The biodiversity monitoring needs “*There is standardised methodology and protocols to ensure consistency*” and “*Mentoring*, *training and support for contributors is provided*” were the two top selections (each was selected 18 times) from the online surveys.

Monitoring needs primarily relating to reporting (i.e. recording), such as “*There are simple ways for everyone to report widespread / common / easily-identified species*” and “*There are systems for electronically capturing data in the field*”, along with “*Examples of best practice are identified and shared between schemes and organisations*” were ranked lowest by the respondents (Table 1). A maximum of 270 top ten selected monitoring needs was possible (27 respondents x 10 monitoring needs), and a total of 266 selections were made. Additional comments on the survey questions, or points considered important, captured from stakeholders are given in S3 File.

There was broad agreement between and within affiliation type for the higher ranking monitoring needs (Table 1), with agreement generally decreasing further down the list. The biodiversity monitoring need: “*There is standardised methodology and protocols to ensure consistency*” was considered to be of importance by Consultants, Government representatives, NGOs and Researchers, with over half the respondents within these affiliations selecting it in their top ten. Biological recorders (i.e. amateur experts) selected a narrower range of monitoring needs that they considered to be of importance, with 13 out of a possible twenty selected; this is in comparison to Consultants and Other expertise who selected 17 and 18 out of 26 respectively.

The two most important themes, with importance being assigned by the number of times they were placed in the top 10, were Design and Human resource (Fig 3). Design, Human resource and Design/Human Resource-based monitoring needs were selected 189 times out of the 266 selections made. Needs classified as Analytical were selected the second most frequently (n = 41). Technological and other (non-human) resource-based needs were viewed as less important by stakeholders.

Overall scoring for the adequacy of the survey in representing biodiversity monitoring gaps in Cyprus was favourable (Table 2). The average score per affiliation-type was over seven (with 10 being most useful). “Governmental” stakeholders agreed most strongly with the monitoring needs (average score 9.2), and the variability in the range of scores was generally low across the stakeholder groups, with the exception of the “Researcher” affiliation that had scores ranging from 1 through to 10. The lowest average score was for the “Researcher” category at 7.4 out of 10.

**Table 2:** Results of stakeholder responses.

Organisation	Average score of usefulness	Range
Biological recorder (n = 2)	8	6–10
Consultant (n = 2)	8	8
Government (n = 7)	9.2	8–10
NGO (n = 5)	7.8	7–10
Researcher (n = 9)	7.4	1–10
Other expertise (n = 2)	8	8

Results of 26 stakeholders responses asked whether the online questionnaire (S1 File) adequately represented any gaps in biological monitoring in Cyprus. Stakeholders were asked to score how useful the survey was from 10 ’very useful’ to 1 ’not useful at all’.

### Step 3. Expert-elicitation workshop

#### 3.i

Twenty-five monitoring needs were taken forward for voting by stakeholders whilst together in plenary, following the merging of two biodiversity monitoring needs, as outlined in the methods above.

#### 3.ii and 3.iii

During the expert-elicitation process that followed the breakout group discussions, the 25 needs were further modified and combined in order to better align with the stakeholders needs or to reduce redundancy within the monitoring needs. The following needs were modified:

1. *“There is standardised methodology and protocols to ensure consistency”*. This was amended to incorporate the following four needs:
  1. *There are suitable field sampling methods that are accurate/efficient*
  2. *There are appropriate analytical/statistical approaches to measure trends from monitoring data*
  3. *Recorders collect supplementary data (such as characteristics of the habitat*, *soil or weather)*. This need was considered important for the marine environment as certain data, e.g. temperature or depth, were considered hard to get for this environment
  4. *Examples of best practice are identified and shared between schemes and organisations*
2. *“Change is reported at appropriate intervals”* and *“Change is reported on an annual basis”* were combined to give the new biodiversity monitoring need “*Change is reported at appropriate intervals e*.*g*. *on an annual basis”*.
3. The biodiversity monitoring need *“There are data systems (e*.*g*. *online) for efficient data capture and storage”* was amended to *“There are improved and accessible data systems (e*.*g*. *online) for efficient data capture and storage”*.

During the expert-elicitation workshop, separate monitoring needs were not assigned individual ranks, but rather consensus was reached on the relative positioning of groups of monitoring needs taken together. The following ranked groups of monitoring needs was established: a top priority group of nine; a middle group of five; and a bottom group of six (Table 3). The relative positions of the monitoring needs from the online survey were very similar to those decided upon during the workshop (accounting for the editing of the six monitoring needs described previously), with monitoring needs broadly being found in the same relative positions in terms of top, middle and lower levels (Table 3). Eight of the top nine biodiversity monitoring needs from the workshop were also in agreement with the results of the online survey (Tables 1 and 3). The largest change in priority for a monitoring need between the online survey and the workshop was “*Communicate the objectives of monitoring*” which increased in relative importance during the workshop, having been selected only nine times during the online scoring exercise but becoming a top need in the workshop.

**Table 3:** Consensus-ranked list of biodiversity monitoring needs.

Theme	Step 3iii List of biodiversity monitoring needs	Consensus priority group
Human resource	Mentoring, training and support for contributors is provided	Top
Design	There is standardised methodology and protocols to ensure consistency	Top
Human resource	There are quality assurance checks undertaken in order to ensure the accuracy of the records	Top
Design	There is national or regional coordination	Top
Human resource	There are sufficient ’and sustained’ contributors with specialist knowledge of their taxa	Top
Technological	There are improved and accessible data systems (e.g. online) for efficient data capture and storage	Top
Human resource	There is sustained participation	Top
Design/Human resource	There is wide coverage across the country/region, e.g. covering remote and well-populated areas	Top
Design	Communicate the objectives of monitoring	Top
Other resource	There are suitable and accessible identification guides	Middle
Analytical	Change is reported at appropriate intervals e.g. on an annual basis	Middle
Design	‘Important’ or ‘indicator’ species have been identified	Middle
Human resource	There is appropriate feedback to participants on survey results and findings	Middle
Analytical	There is access to analytical expertise to measure trends from monitoring data	Middle
Design/Human resource	There is extra effort on protected areas, e.g. Natura 2000 sites	Bottom
Design	There is a scientific scheme design (such as stratified or randomised site selection) for statistical rigour	Bottom
Design/Human resource	There is extra effort on priority species and habitats	Bottom
Technological/Human resource	The data from monitoring schemes are widely disseminated	Bottom
Design/Technological	There are simple ways for everyone to report widespread/common/easily-identified species [wider engagement]	Bottom
Technological	There are systems for electronically capturing data in the field	Bottom

Consensus-ranked list of biodiversity monitoring needs from the expert-elicitation workshop (Step 3), held in Cyprus in August 2017. Thirty-nine stakeholders participated in the workshop from the field of biodiversity monitoring in Cyprus and the UK. The number of votes each monitoring need received in the online survey was given and was used as a method for initially ranking the needs at the start of the elicitation work. During the expert-elicitation process, five (of the 25) biodiversity monitoring needs were combined with to generate a final list of 20 for Cyprus. Consensus was reached on the top nine, the middle five and the bottom six monitoring needs. *Themes were added post-workshop.

## Discussion

Globally there is a need to deliver robust data to quantify changes in biodiversity, thereby helping to ensure that conservation funds and other resources are used optimally. This goal, however, also requires an appropriate prioritisation strategy wherever resources are limited, e.g. in a given region should we focus on training volunteers or employing more professionals? Should we record broad-scale distributions across multiple taxa, or intensively monitor a small selection of species’ populations? To assist with this goal, we have developed a structured set of biodiversity monitoring needs for Cyprus using questionnaire-based online rankings and subsequent workshop-based expert-elicitation. High priority needs in Cyprus primarily focused around two themes: Human resource and Design. Despite technologies playing an important role in advancing the generation and utilisation of biological data for conservation in some locations [57], we found that technology-based needs were a lower priority for our stakeholders. Needs related to data analysis ranked in the middle of both our online and expert-elicitation rankings.

Monitoring needs focused on design were prioritised highly, but those needs focused on human resources, such as:

- “*Mentoring*, *training and support for contributors is provided*”,
- “*There are quality assurance checks undertaken in order to ensure the accuracy of the records*” and
- “*There are sufficient ’and sustained’ contributors with specialist knowledge of their taxa*”, were also important.

Volunteer enthusiasm is known to be a key driver in sustaining participation in biodiversity recording schemes [44], and this can be supported through mentoring and training [58].

Expert-elicitation, as a mechanism for the development of a prioritised list of biodiversity monitoring needs in Cyprus, was selected following the successful application of this technique in the UK [44]. Indeed, Moreira and others [59] also recommend that alongside increasing transdisciplinary research, the identification of research priorities needs to be inclusive of the range of stakeholder interests, as well as regional needs and possible funding mechanisms. However, despite the many recorded benefits of stakeholder involvement, this approach to elucidating answers can also risk bringing in participant bias [40]. For example, within our workshop, although 27 stakeholders took part in the online survey and 39 stakeholders in the workshop, the biological recording community (i.e. amateur experts, or citizen scientists collecting biological records) was arguably under-represented, which may have shifted the focus from the prioritisation of results towards data user needs, as data contributors can have different motivations to users. The stakeholders participating in both the online questionnaire and expert-elicitation workshop were primarily end-users of data; however they did not solely prioritise needs focused on design and analysis (professional end users typically rely on detailed and accurate systematic data to inform decision-making). This could be attributed to limitations inherent to one often encountered definition of data users: data users are traditionally considered to be academics or government agencies [44]. Whilst this assumption may hold true in some places and at some times, it may also be the case that data users are contributors of biodiversity data. Those stakeholders who are also undertaking recording in a voluntary capacity may therefore have a more human resource-focused view of monitoring needs. As such, through this expert-elicitation process, we suggest that we have reached a reasonably robust community consensus on the biodiversity monitoring needs most relevant to Cyprus.

It was unexpected that technology-based needs, and needs associated with technology, were not ranked consistently highly in both the online questionnaire and workshop. Advances in technology, for example websites that allow for rapid data entry, that promote straightforward data sharing, or which facilitate the automation of record “validation”, are an important part of modern biological recording [57]. However, the ready availability of some technologies at this point in history, such as mobile apps and data sharing portals, are likely to mean that these biodiversity monitoring needs are not as important when compared to having enough human resources (support and mentoring etc.) to undertake the monitoring in the first instance.

Reporting back to data collectors is often seen as a key motivator for ensuring engagement of recorders (e.g. see 24). However the biodiversity monitoring need “*The data from monitoring schemes are widely disseminated*” was one of the lowest priorities for both the online questionnaire and expert-elicitation workshop. Its placement could be due to other biodiversity monitoring needs being considered higher priorities, rather than this aspiration being unimportant per se. One stakeholder suggested that this could be due to local scientific monitoring cultures in place on the island that resist data sharing due to perceptions relating to the loss of control over such data, and/or the lack of professional reward currently associated with such actions.

The successful adaptation of the framework of Pocock and others [44] in this exercise supports their contention that the framework was replicable and robust for other countries. With respect to the prioritisation of the biodiversity monitoring needs, our results broadly reflected the findings of Pocock and others [44], with both the online and expert-elicitation workshop prioritising standard methodologies, quality assurance, and suitable field sampling methods that are accurate and efficient. Our findings did however deviate in the increased prioritisation of needs relating to national/regional coordination, and the collection of additional environmental data, such as climatic conditions and additional data for the marine environment; these were both listed in the top nine biodiversity monitoring needs group in the current study (they were amongst the least important in Pocock and others [44]). The collection of additional environmental data, such as habitat information, can increase the value of records in answering ecological questions [60]. Their increased importance here may in part be due to concern over the impacts of climate change on biodiversity, as the Mediterranean is predicted to be particularly strongly affected through climatic conditions such as drought and high temperatures [61, 62]. The specific reference to collecting data in the marine environment is likely linked to both the lack of data in many taxonomic groups of marine organisms and the lack of available marine environmental data in Cyprus. The Mediterranean Sea is facing multiple pressures from climate change, overexploitation of marine resources and invasive alien species [8, 63], and, as such, additional data to monitor and record change is very important for this vulnerable system.

### Possible uses and next steps for the prioritised list of biodiversity monitoring needs

#### Informing the design of monitoring schemes

Pocock and others [44] recommend using such prioritised lists to look for gaps in existing programmes, and also in the design of new biodiversity monitoring programmes. We found that the most highly prioritised monitoring needs were those related to the development of robust methodologies and in ensuring that sufficiently skilled citizens are available to contribute (including being available to record across wide geographic regions). These prioritised needs could be used to help focus existing resources for data collection. For example, supporting the development of standardised methodologies would enable structured biological recording [28], thus ensuring consistency in the way records are collected in new schemes. This would also support recognition of the importance of mentoring, training and supporting volunteers in existing, or newly establishing, schemes which could result in increased funding for these activities [57].

#### Increasing infrastructure capacity

National coordination was also considered to be a top biodiversity monitoring need (Table 3). Although there are many biodiversity datasets within Cyprus, there are gaps in collating, analysing, identifying trends and needs, and communicating and sharing data. There would be merit in developing guidance on mechanisms for extracting data from a selection of sources (e.g. from studies undertaken for another purpose, but that include biodiversity data), how to manage this information (e.g. existence of appropriate software and training to use it), and how to analyse, interpret, and share these among interested parties [13].

Establishing a national hub for the centralised collation, dissemination and analysis of biodiversity monitoring data could help to deliver the data needed to enable governments to report on environmental change, and help to prioritise suitable monitoring approaches for sites or species that need protecting, or for which monitoring is specifically mandated by law [64]. A national hub holding high quality, verified biological records could also play a role in coordinating and supporting a network of citizen science recorders, a model successfully employed in the UK for over 50 years [64]. Such a national hub was suggested from the online survey by one respondent (S3 File) and could be a valuable tool in delivering knowledge about biodiversity in Cyprus. Developing a GBIF Participant node could help enable the coordination of networks of people and institutions that create, manage and utilise data on biodiversity across Cyprus. However, there can be mistrust from data users over the quality of the data in large aggregated datasets, such as inconsistent taxonomy, inadequate spatial coverage, or lack of reported protocols [65], which can lead to reduction in the use of the data for scientific studies [66]. Franz and Sterner [66] also argue for greater accountability of the role of these databases in making choices that directly underpin the perceived quality of aggregated biodiversity data, such as the creation and maintenance of taxonomic hierarchies that may not reflect the concepts used in local biodiversity communities. Franz and Sterner [66] call for global data hubs to work to develop new technical pathways and social incentives as mechanisms to bridge the current trust gap.

#### Developing cross-taxa networks

“*Examples of best practice are identified and shared between schemes and organisations*” was not highly scored during the online questionnaire (S1 File). During the expert-elicitation workshop, this biodiversity monitoring need was subsequently amalgamated with three other needs:

“*There are suitable field sampling methods that are accurate/efficient*”;

“*There are appropriate analytical/statistical approaches to measure trends from monitoring data*”; and

“*Recorders collect supplementary data (such as characteristics of the habitat*, *soil or weather)*” to give the biodiversity monitoring need “*There is standardised methodology and protocols to ensure consistency*”. This became one of the top nine biodiversity monitoring needs. Should this need become a higher actual priority on Cyprus, the approach taken by the National Forum for Biological Recording (NFBR) charity in the UK may be relevant. The NFBR works across taxonomic groups to support biological recorders, hosts conferences, and represents their interests in governmental decision-making, through mechanisms such as the State of Nature report [67]. Such a model could enable the Cypriot biological recording community to share ideas and experiences for the maintenance and support of biodiversity monitoring.

## Conclusion

Prioritising biodiversity monitoring needs is becoming increasingly important as we continue to see major declines in biodiversity and degradation of ecosystem function [1]. Through a consensus exercise, a hierarchy of three groups of monitoring needs was created. As well as the priority list supporting decision-making by providing a snapshot of one consensus developed across a range of biodiversity monitoring stakeholders, we hope that the full list of needs will also assist with the development of robust monitoring programmes simply by providing a checklist of considerations. In this way, organisations and funders can ensure that all relevant monitoring needs have been considered, and that the, inevitably values-based, prioritisation that must take place whenever funds are limited is based on a full, and hopefully transparent, consideration of all available options.

## Supporting information

S1 File Online survey questionnaire. English version of online survey questionnaire sent to stakeholders working in the field of biodiversity monitoring in Cyprus (in both Greek and English). Results were collected using the online survey platform https://www.onlinesurveys.ac.uk/.
(DOCX)

S2 File Unprocessed results from an online survey. Unprocessed results from an online survey sent to stakeholders working in the field of biodiversity monitoring in Cyprus (in both Greek and English). Information that would make individuals identifiable has been removed for the purposes of publication. Each respondent has a unique code generated by the online survey. Respondents were asked if they were able to attend the workshop. They were also asked to select their organisation from a pre-populated list or to select “other” and then specify. Responses that were submitted in Greek were translated by the project partners. Respondents were then asked to give their top three biodiversity monitoring interests from a pre-populated list with a free text box for additional information. Respondents were then asked to rank biodiversity monitoring needs given in a pre-populated list that represent the 10 most important gaps or opportunities in biological recording. Next, stakeholders were asked to rank how adequately the questionnaire addressed the biological recording gaps in Cyprus. Finally, stakeholders were asked if they wished to be co-authors on the current manuscript.
(XLSX)

S3 File Additional stakeholder comments from both an online questionnaire and workshop. Additional stakeholder comments from both an online questionnaire and workshop involving stakeholders from the field of biodiversity monitoring in Cyprus. Stakeholders were asked to give further features or comments that they considered important and that might have missed out from either the online survey or that came up as part of the discussions. A facilitator summarised the workshop discussion points. This task was undertaken as part of a wider exercise on ranking the biodiversity monitoring needs for Cyprus. *Themes were added post-workshop.
(DOCX)

## References

[1] Díaz S, Settele J, Brondízio E, Hien N, M G, Agard J, et al. Summary for policymakers of the global assessment report on biodiversity and ecosystem services of the Intergovernmental Science-Policy Platform on Biodiversity and Ecosystem Services. IPBES, 2019 6 May 2019. Report No.

[2] Erotokritou E, Vogiatzakis IN. Landscape linkages for the distribution of the endangered Hierophis cypriensis in Cyprus. Ecologia mediterranea: Revue internationale d’écologie méditerranéenne = International Journal of Mediterranean Ecology. 2019;45(1):31–44.

[3] Sala OE, Chapin FStuart III, Armesto JJ, Berlow E, Bloomfield J, et al. Global Biodiversity Scenarios for the Year 2100. Science. 2000;287(5459):1770–4. doi: 10.1126/science.287.5459.1770 10710299

[4] Coll M, Piroddi C, Steenbeek J, Kaschner K, Lasram FBR, Aguzzi J, et al. The biodiversity of the Mediterranean Sea: estimates, patterns, and threats. PloS one. 2010;5(8):e11842. doi: 10.1371/journal.pone.0011842 20689844 PMC2914016

[5] Hall CM. Tourism and biodiversity: more significant than climate change? Journal of Heritage Tourism. 2010;5(4):253–66. doi: 10.1080/1743873X.2010.517843

[6] Sánchez-Bayo F, Wyckhuys KAG. Worldwide decline of the entomofauna: A review of its drivers. Biol Conserv. 2019;232:8–27. doi: 10.1016/j.biocon.2019.01.020

[7] Nieto A, Roberts SP, Kemp J, Rasmont P, Kuhlmann M, Criado MG, et al. European red list of bees. 2017.

[8] Tsikliras AC, Dinouli A, Tsiros V-Z, Tsalkou E. The Mediterranean and Black Sea Fisheries at Risk from Overexploitation. PLOS ONE. 2015;10(3):e0121188. doi: 10.1371/journal.pone.0121188 25793975 PMC4368760

[9] McNeely JA. The sinking ark: pollution and the worldwide loss of biodiversity. Biodiversity & Conservation. 1992;1(1):2–18.

[10] Defra. A Green Future: Our 25 Year Plan to Improve the Environment. Defra, 2018.

[11] Gitzen RA, Millspaugh JJ, Cooper AB, Licht DS. Design and analysis of long-term ecological monitoring studies: Cambridge University Press; 2012.

[12] Hochkirch A, Samways MJ, Gerlach J, Böhm M, Williams P, Cardoso P, et al. A strategy for the next decade to address data deficiency in neglected biodiversity. Conserv Biol. 2020. doi: 10.1111/cobi.13589 32656858

[13] Groom QJ, Desmet P, Vanderhoeven S, Adriaens T. The importance of open data for invasive alien species research, policy and management. Management of Biological Invasions. 2015;6(2):119.

[14] Beck J, Ballesteros-Mejia L, Nagel P, Kitching IJ. Online solutions and the ‘Wallacean shortfall’: what does GBIF contribute to our knowledge of species’ ranges? Divers Distrib. 2013;19(8):1043–50. doi: 10.1111/ddi.12083

[15] Brown J, Lomolino M. Biogeography, 2nd edn Sinauer Associates: Sunderland. MA, USA. 1998.

[16] Brito D. Overcoming the Linnean shortfall: Data deficiency and biological survey priorities. Basic and Applied Ecology. 2010;11(8):709–13. doi: 10.1016/j.baae.2010.09.007

[17] de Carvalho MR, Bockmann FA, Amorim DS, Brandão CRF, de Vivo M, de Figueiredo JL, et al. Taxonomic Impediment or Impediment to Taxonomy? A Commentary on Systematics and the Cybertaxonomic-Automation Paradigm. Evolutionary Biology. 2007;34(3):140–3. doi: 10.1007/s11692-007-9011-6

[18] Kim KC, Byrne LB. Biodiversity loss and the taxonomic bottleneck: emerging biodiversity science. Ecological Research. 2006;21(6):794.

[19] Boxshall G, Self D. UK Taxonomy & Systematics Review–2010. Results of survey undertaken by the Review Team at the Natural History Museum serving as contractors to the Natural Environment Research Council (NERC). 2011.

[20] Agnarsson I, Kuntner M. Taxonomy in a changing world: seeking solutions for a science in crisis. Systematic Biology. 2007;56(3):531–9. doi: 10.1080/10635150701424546 17562477

[21] Hochkirch A. The insect crisis we can’t ignore. Nature. 2016;539(7628):141-. doi: 10.1038/539141a 27830820

[22] Juanes F. Visual and acoustic sensors for early detection of biological invasions: Current uses and future potential. Journal for Nature Conservation. 2018;42:7–11. doi: 10.1016/j.jnc.2018.01.003

[23] Piaggio AJ, Engeman RM, Hopken MW, Humphrey JS, Keacher KL, Bruce WE, et al. Detecting an elusive invasive species: a diagnostic PCR to detect Burmese python in Florida waters and an assessment of persistence of environmental DNA. Molecular ecology resources. 2014;14(2):374–80. doi: 10.1111/1755-0998.12180 24119154

[24] Morris R. A change in funding directions: Implications for biological recording. British Journal of Entomology and Natural History. 2012;25(3):143.

[25] Isaac NJB, Pocock MJO. Bias and information in biological records. Biological Journal of the Linnean Society. 2015;115(3):522–31. doi: 10.1111/bij.12532

[26] Kampen H, Medlock JM, Vaux AGC, Koenraadt CJM, van Vliet AJH, Bartumeus F, et al. Approaches to passive mosquito surveillance in the EU. Parasites & Vectors. 2015;8(1):9. doi: 10.1186/s13071-014-0604-5 25567671 PMC4302443

[27] Preston CD. Following the BSBI’s lead: the influence of the Atlas of the British flora, 1962–2012. New Journal of Botany. 2013;3(1):2–14. doi: 10.1179/2042349713Y.0000000020

[28] Pescott OL, Walker KJ, Pocock MJ, Jitlal M, Outhwaite CL, Cheffings CM, et al. Ecological monitoring with citizen science: the design and implementation of schemes for recording plants in Britain and Ireland. Biological Journal of the Linnean Society. 2015;115(3):505–21.

[29] Pocock MJO, Roy HE, Preston CD, Roy DB. The Biological Records Centre: a pioneer of citizen science. Biological Journal of the Linnean Society. 2015;115(3):475–93. doi: 10.1111/bij.12548

[30] Brereton T, Botham M, Middlebrook I, Randle Z, Noble D, Roy D. United Kingdom Butterfly Monitoring Scheme Annual Report 2016. 2017.

[31] Comont R, Miles S. BeeWalk Annual Report 2019. Bumblebee Conservation Trust, Stirling, Scotland UK. 2019.

[32] CIESM ICfSEotMS. CIESM JellyWatch Program 2014 [cited 2021 12/04/2021]. http://www.ciesm.org/marine/programs/jellywatch.htm.

[33] Crall AW, Renz M, Panke BJ, Newman GJ, Chapin C, Graham J, et al. Developing cost-effective early detection networks for regional invasions. Biol Invasions. 2012;14(12):2461–9. doi: 10.1007/s10530-012-0256-3

[34] Giovos I, Kleitou P, Poursanidis D, Batjakas I, Bernardi G, Crocetta F, et al. Citizen-science for monitoring marine invasions and stimulating public engagement: a case project from the eastern Mediterranean. Biol Invasions. 2019;21(12):3707–21.

[35] Groom Q, Strubbe D, Adriaens T, Davis AJ, Desmet P, Oldoni D, et al. Empowering Citizens to Inform Decision-Making as a Way Forward to Support Invasive Alien Species Policy. Citizen Science: Theory and Practice. 2019;4(1).

[36] Kleitou P, Giovos I, Wolf W, Crocetta F. On the importance of citizen-science: the first record of Goniobranchus obsoletus (Rüppell and Leuckart, 1830) from Cyprus (Mollusca: Gastropoda: Nudibranchia). BioInvasions Records. 2019;8(2):252–7.

[37] Wetzel FT, Bingham HC, Groom Q, Haase P, Kõljalg U, Kuhlmann M, et al. Unlocking biodiversity data: Prioritization and filling the gaps in biodiversity observation data in Europe. Biol Conserv. 2018;221:78–85.

[38] García-Barón I, Giakoumi S, Santos MB, Granado I, Louzao M. The value of time-series data for conservation planning. J Appl Ecol. 2021.

[39] Colson AR, Cooke RM. Expert Elicitation: Using the Classical Model to Validate Experts’ Judgments. Review of Environmental Economics and Policy. 2018;12(1):113–32. doi: 10.1093/reep/rex022

[40] Sutherland WJ, Burgman M. Policy advice: use experts wisely. Nature News. 2015;526(7573):317. doi: 10.1038/526317a 26469026

[41] Roy HE, Peyton JM, Booy O. Guiding principles for utilizing social influence within expert-elicitation to inform conservation decision-making. Glob Change Biol. 2020;n/a(n/a). doi: 10.1111/gcb.15062 32227619

[42] Sutherland WJ, Dias MP, Dicks LV, Doran H, Entwistle AC, Fleishman E, et al. A horizon scan of emerging global biological conservation issues for 2020. Trends Ecol Evol. 2020;35(1):81–90. doi: 10.1016/j.tree.2019.10.010 31813647

[43] Grace M, Balzan M, Collier M, Geneletti D, Tomaskinova J, Abela R, et al. Priority knowledge needs for implementing nature-based solutions in the Mediterranean islands. Environmental Science & Policy. 2021;116:56–68. doi: 10.1016/j.envsci.2020.10.003

[44] Pocock MJ, Newson SE, Henderson IG, Peyton J, Sutherland WJ, Noble DG, et al. Developing and enhancing biodiversity monitoring programmes: a collaborative assessment of priorities. J Appl Ecol. 2015;52(3):686–95. doi: 10.1111/1365-2664.12423 27642189 PMC5008152

[45] CBD CoBD. Fourth National Report to the United Nations Convention on Biological Diversity. Nicosia: Department of Environment—Ministry of Agriculture, Natural Resources and Environment, 2010.

[46] Delipetrou P, Makhzoumi J, Dimopoulos P, Georghiou K. Cyprus. Mediterranean Island Landscapes: Springer; 2008. p. 170–203.

[47] Vogiatzakis IN, Manolaki P, Zomeni M, Zotos S. Habitats. In: Sparrow DJ, John E, editors. An introduction to the wildlife of Cyprus: Terra Cypria; 2016.

[48] Manolaki P, Zotos S, Vogiatzakis IN. An integrated ecological and cultural framework for landscape sensitivity assessment in Cyprus. Land Use Policy. 2020;92:104336. doi: 10.1016/j.landusepol.2019.104336

[49] Varnava AI, Roberts SP, Michez D, Ascher JS, Petanidou T, Dimitriou S, et al. The wild bees (Hymenoptera, Apoidea) of the island of Cyprus. ZooKeys. 2020;924:1. doi: 10.3897/zookeys.924.38328 32308528 PMC7154044

[50] Sparrow DJ, John E. An introduction to the wildlife of Cyprus: Terra Cypria; 2016.

[51] Christodoulou CS. The impact of Acacia saligna invasion on the autochthonous communities of the Akrotiri salt marshes. University of Central Lancashire, Preston. 2003.

[52] Tsintides T, Christodoulou CS, Delipetrou P, Georghiou K. The red data book of the flora of Cyprus. Cyprus Forestry Association Lefkosia 465. 2007.

[53] Termaat T, van Strien AJ, van Grunsven RHA, De Knijf G, Bjelke U, Burbach K, et al. Distribution trends of European dragonflies under climate change. Divers Distrib. 2019;25(6):936–50. doi: 10.1111/ddi.12913

[54] Sevilleja C, Collins S, Warren M, Wynhoff I, Van Swaay C, Dennis E, et al. European Butterfly Monitoring Scheme (eBMS): network development. Technical report. Butterfly Conservation Europe and ABLE/eBMS, 2020.

[55] Hand R, Hadjikyriakou GN, Christodoulou CS. (continuously updated): Flora of Cyprus—a dynamic checklist. Published at http://www.flora-of-cyprus.eu/2011-2020 [cited 2020 09/10/2020]. http://www.flora-of-cyprus.eu/.

[56] Roy HE, Peyton J, Aldridge DC, Bantock T, Blackburn TM, Britton R, et al. Horizon scanning for invasive alien species with the potential to threaten biodiversity in Great Britain. Glob Change Biol. 2014;20(12):3859–71. doi: 10.1111/gcb.12603 24839235 PMC4283593

[57] August T, Harvey M, Lightfoot P, Kilbey D, Papadopoulos T, Jepson P. Emerging technologies for biological recording. Biological Journal of the Linnean Society. 2015;115(3):731–49.

[58] Bell S, Marzano M, Cent J, Kobierska H, Podjed D, Vandzinskaite D, et al. What counts? Volunteers and their organisations in the recording and monitoring of biodiversity. Biodivers Conserv. 2008;17(14):3443–54. doi: 10.1007/s10531-008-9357-9

[59] Moreira F, Allsopp N, Esler KJ, Wardell-Johnson G, Ancillotto L, Arianoutsou M, et al. Priority questions for biodiversity conservation in the Mediterranean biome: Heterogeneous perspectives across continents and stakeholders. Conservation Science and Practice. 2019;1(11):e118. doi: 10.1111/csp2.118

[60] Sutherland WJ, Roy DB, Amano T. An agenda for the future of biological recording for ecological monitoring and citizen science. Biological Journal of the Linnean Society. 2015;115(3):779–84. doi: 10.1111/bij.12576

[61] Giannakopoulos C, Hadjinicolaou P, Kostopoulou E, Varotsos K, Zerefos C. Precipitation and temperature regime over Cyprus as a result of global climate change. Advances in Geosciences. 2010;23:17–24.

[62] Hadjinicolaou P, Giannakopoulos C, Zerefos C, Lange MA, Pashiardis S, Lelieveld J. Mid-21st century climate and weather extremes in Cyprus as projected by six regional climate models. Regional Environmental Change. 2011;11(3):441–57.

[63] Kleitou P, Crocetta F, Giakoumi S, Giovos I, Hall-Spencer JM, Kalogirou S, et al. Fishery reforms for the management of non-indigenous species. Journal of Environmental Management. 2021;280:111690. doi: 10.1016/j.jenvman.2020.111690 33246748

[64] Roy HE, Preston CD, Roy DB. Fifty years of the Biological Records Centre. Biological Journal of the Linnean Society. 2015;115(3):469–74. doi: 10.1111/bij.12575

[65] Franklin J, Serra-Diaz JM, Syphard AD, Regan HM. Big data for forecasting the impacts of global change on plant communities. Glob Ecol Biogeogr. 2017;26(1):6–17. doi: 10.1111/geb.12501

[66] Franz NM, Sterner BW. To increase trust, change the social design behind aggregated biodiversity data. Database. 2018;2018. doi: 10.1093/database/bax100 29315357 PMC7206650

[67] Hayhow D, Eaton M, Stanbury A, Burns F, Kirby W, Bailey N, et al. State of nature 2019. 2019.

