# Supplementary material for: Using expert-elicitation to deliver biodiversity monitoring priorities on a Mediterranean island
Source: PLoS One. 2022 Mar 24;17(3):e0256777. doi: 10.1371/journal.pone.0256777 (PMC8947143; doi:10.1371/journal.pone.0256777)
Supplement: S1 File — English version of online survey questionnaire sent to stakeholders working in the field of biodiversity monitoring in Cyprus (in both Greek and English). Results were collected using the online survey platform https://www.onlinesurveys.ac.uk/. (DOCX) [file pone.0256777.s001.docx]

# Supplementary Information 1. Online survey questionnaire sent to stakeholders working in the field of biodiversity monitoring in Cyprus (in both Greek and English). Results were collected using the online survey platform <https://www.onlinesurveys.ac.uk/>.

Capacity Building in Monitoring and Surveillance of native and non-native species

Page 1: About Us

The RIS-Ký (Researching Invasive Species of Kýpros) project started in April 2017 looking at the impacts of invasive alien species in terrestrial, freshwater and marine habitats on Sovereign Base Areas in Cyprus (SBAs).

RIS-Ký is a collaboration between the UK Centre for Ecology &Hydrology (www.ceh.ac.uk) in the UK, the Joint Services Health Unit, Cyprus and the University of Cyprus.

RIS-Ký will run for two years and is funded by the Darwin Initiative. Please visit our website if you like to know more [www.ris-ky.info](http://www.ris-ky.info).

In April 2017 we hosted a very successful and productive workshop on sharing invasive alien species information and horizon scanning at the Akrotiri Environmental Education Centre in April 2017 involving many experts from government departments, universities, research organizations and NGOs from Cyprus, the UK, Greece, Israel, Belgium and Italy. A list of invasive alien species most likely to be introduced to Cyprus within the next 10 years was agreed. Following on from this exercise, we are now looking to understand more of the needs of the SBAs, but also island wide, regarding biological recording and the potential role of citizen science and public engagement in fulfilling biological recording and monitoring for biodiversity (with a focus on alien species). Therefore, we are extremely keen to know your thoughts regarding the current status and future priorities for biological recording.

We are very much hoping you are able to attend our workshop on 31 of August. In preparation for this workshop (and even if you are unable to attend) we would be grateful if you can fill in this short questionnaire on the biological recording in Cyprus and SBAs. The results of the questionnaire will be discussed during our workshop and subsequently developed within a manuscript to which you are invited to be co-authors if you wish so.

This survey should take no more than 15 minutes.

Please come back to us if you have any questions about this survey info@ris-ky.info.

1. Please indicate below whether you are able to attend our workshop on 31st August 2017

Yes / No

1. Please give your name, affiliation (organization) and email address if you are able to attend.

2a. Organisation

- 1. NGO
  2. Government
  3. University
  4. Research institute
  5. Biological recording scheme
  6. Other (please give info below)

1. Please give your top three monitoring / recording priorities. These can either be taxonomic specific (e.g. monitoring invasive species, birds or butterflies), or environment focused (e.g. monitoring the water levels in the Akrotiri Lake).

3.1. Lichens

3.2. Bryophytes

3.3. Plants

3.4. Insect invertebrates

3.5. Non-insect invertebrates

3.6. Amphibians and reptiles

3.7. Birds

3.8 Mammals

3.9. No specific expertise

3.10. Other expertise

Please feel free to share extra information on your taxa

1. We suggest you make this a two-stage process. Please select 'N/A' for the statements that you consider as least important to you, until you are left with 10. We recognise that many people will think that most of the statements are important, but we would like you to leave only your top 10 to help us identify priorities for supporting biological recording.)

4.1. Communicate the objectives of monitoring;

4.2. There is standardised methodology and protocols to ensure consistency;

4.3. There are suitable field sampling methods that are accurate/efficient;

4.4. There are sufficient contributors;

4.5. There are suitable and accessible identification guides;

4.6. There is national or regional co-ordination;

4.7. There are data systems (e.g. online) for efficient data capture and storage;

4.8. There are quality assurance checks undertaken in order to ensure the accuracy of the records;

4.9. There is appropriate feedback to participants on survey results and findings;

4.10. There are sufficient contributors with specialist knowledge of their taxa;

4.11. There are appropriate analytical/statistical approaches to measure trends from monitoring data;

4.12. There is sustained participation;

4.13. Mentoring, training and support for contributors is provided;

4.14. There is access to analytical expertise to measure trends from monitoring data;

4.15. Change is reported at appropriate intervals;

4.16. There is a scientific scheme design (such as stratified or randomised site selection) for statistical rigour;

4.17. There are simple ways for everyone to report widespread/common/easily-identified species;

4.18. The data from monitoring schemes are widely disseminated;

4.19. Examples of best practice are identified and shared between schemes and organisations;

4.20. ‘Important’ or ‘indicator’ species have been identified;

4.21. There is wide coverage across the country/region, e.g. covering remote and well-populated areas;

4.22. Recorders collect supplementary data (such as characteristics of the habitat, soil or weather);

4.23. There is extra effort on priority species and habitats;

4.24. There is extra effort on protected areas, e.g. Natura 2000 sites;

4.25. There are systems for electronically capturing data in the field;

4.26. Change is reported on an annual basis.

Please add in the box below, any further features/comments that you consider important and we might have missed out.

4.b. Did you think that this questionnaire adequately represents any gaps in biological recording in Cyprus? Please score how useful you think it was from 10 'very useful' to 1 'not useful at all'.

4.c. Please indicate whether you want to be a co-author to the subsequent manuscript.
